# Model Transferability and Reduced Experimental Burden in Cell Culture Process Development Facilitated by Hybrid Modeling and Intensified Design of Experiments

**DOI:** 10.3389/fbioe.2021.740215

**Published:** 2021-12-23

**Authors:** Benjamin Bayer, Mark Duerkop, Gerald Striedner, Bernhard Sissolak

**Affiliations:** ^1^ Department of Biotechnology, University of Natural Resources and Life Sciences, Vienna, Austria; ^2^ Novasign GmbH, Vienna, Austria; ^3^ Bilfinger Life Science GmbH, Salzburg, Austria

**Keywords:** Chinese hamster ovary cells, quality by design, intensified DoE, monoclonal antibody, upstream processing, grey box, scale up, process optimization

## Abstract

Reliable process development is accompanied by intense experimental effort. The utilization of an intensified design of experiments (iDoE) (intra-experimental critical process parameter (CPP) shifts combined) with hybrid modeling potentially reduces process development burden. The iDoE can provide more process response information in less overall process time, whereas hybrid modeling serves as a commodity to describe this behavior the best way. Therefore, a combination of both approaches appears beneficial for faster design screening and is especially of interest at larger scales where the costs per experiment rise significantly. Ideally, profound process knowledge is gathered at a small scale and only complemented with few validation experiments on a larger scale, saving valuable resources. In this work, the transferability of hybrid modeling for Chinese hamster ovary cell bioprocess development along process scales was investigated. A two-dimensional DoE was fully characterized in shake flask duplicates (300 ml), containing three different levels for the cultivation temperature and the glucose concentration in the feed. Based on these data, a hybrid model was developed, and its performance was assessed by estimating the viable cell concentration and product titer in 15 L bioprocesses with the same DoE settings. To challenge the modeling approach, 15 L bioprocesses also comprised iDoE runs with intra-experimental CPP shifts, impacting specific cell rates such as growth, consumption, and formation. Subsequently, the applicability of the iDoE cultivations to estimate static cultivations was also investigated. The shaker-scale hybrid model proved suitable for application to a 15 L scale (1:50), estimating the viable cell concentration and the product titer with an NRMSE of 10.92% and 17.79%, respectively. Additionally, the iDoE hybrid model performed comparably, displaying NRMSE values of 13.75% and 21.13%. The low errors when transferring the models from shaker to reactor and between the DoE and the iDoE approach highlight the suitability of hybrid modeling for mammalian cell culture bioprocess development and the potential of iDoE to accelerate process characterization and to improve process understanding.

## 1 Introduction

Usually, tremendous effort is spent performing design of experiments (DoE) as part of process characterization and optimization (Hakemeyer et al., 2016; Tripathi and Shrivastava, 2019; Bayer et al., 2020b) at different scales. This leads to a huge burden on process development and enormous consumption of resources. Depending on the dimensionality of the design space, the burden can increase drastically. Upstream processes in general and in particular mammalian cell culture cultivations can be considered highly complex processes. Several factors, referred to as critical process parameters (CPPs), can have an impact on the produced quantity of the target molecule and certain critical quality attributes (CQAs). This impact is either directly due to changes in the surrounding environment or indirectly due to altering how cells are producing the target molecules.

Especially in biopharmaceutical production, the CQAs have to be within a narrow range to ensure patient safety. Hence, in consideration of the quality-by-design (QbD) approach (ICH Harmonised Tripartite Guideline, 2009), this very impact of the CPP on the CQA needs to be identified and understood. This is achieved by utilizing a certain DoE approach. By performing a DoE, data are generated and CPP effects can be studied. Since each of these approaches has their intrinsic strengths and weaknesses, the provided information also varies significantly (Kumar et al., 2014).

The full-factorial characterization is the most common design for design spaces with less than four parameters due to its simplicity, resulting in high experimental effort. However, several years ago, the concept of reduced experimental effort had already been suggested, and recent advances showed that a similar output with minimal loss in information can be gained with a reduced experimental burden by applying intensified DoE (iDoE) (von Stosch and Willis, 2016; Sommeregger et al., 2017). It was demonstrated that for an *Escherichia coli* fed-batch process, up to 67% of experiments can be spared by using an iDoE approach for process characterization instead of the standard DoE (Bayer et al., 2020b). This iDoE concept is based on introducing intra-experimental process variations (Spadiut et al., 2013). Herein, by performing intra-experimental CPP shifts, the characterization of multiple CPP combinations within one experiment is possible, accelerating the characterization of a design space. Additionally, these shifts induce changes in the specific rates (e.g., growth, consumption, and formation), maximizing the information output and enhancing process understanding. In another publication, it was shown that the iDoE approach was able to provide an in-depth process understanding of a mammalian cell culture fed-batch process (Pappenreiter et al., 2019).

Bioprocess modeling offers the possibility of exploiting the information resulting from a DoE, e.g., to understand the process behavior. Such models can then further be used to establish proper tools for process monitoring and control to keep the CQAs within their narrow operating ranges (Smiatek et al., 2020). Generally, three different modeling approaches are utilized, namely, mechanistic modeling, statistical modeling by multivariate data analysis (MVDA), or hybrid modeling approaches. A detailed description, evaluation, and comparison of these modeling approaches have already been reported (Solle et al., 2017). Considering a mammalian upstream process, a pure mechanistic modeling approach takes high effort to develop due to the complex nature of such a process. Currently, the MVDA approaches used comprise partial least squares regression, artificial neural networks, and support vector machines (Kadlec et al., 2009), and novel applications constantly increase the applicability of MVDA (Clavaud et al., 2013; Mercier et al., 2014; Sokolov et al., 2015).

Such established MVDA models might not be flexible to deal with process setup changes and often have limited extrapolation capabilities. Process scaling, for instance from a shake flask to a bioreactor, commonly results in differences in how the CPPs affect the CQAs and potentially the cell behavior itself (Fox et al., 2004; Li et al., 2010; Sissolak et al., 2019). It might even be possible that other parameters, which were not showing any impact at all, are now significantly altering the CQAs when changing the scale. CO_2_ removal, oxygen transfer, and concentration gradients in a stirred tank bioreactor are such parameters, which are not considered relevant in a small-scale shaker dataset (Xing et al., 2009; Nadal-Rey et al., 2021). The more knowledge about a certain process is available, the fewer experiments have to be performed when hybrid modeling approaches are chosen to develop a well-performing model (Yang et al., 2015; Bayer et al., 2020c; Smiatek et al., 2020). Recently, the incorporation of mechanistic understanding at different levels in different hybrid modeling approaches was evaluated for chromatography (Narayanan et al., 2021a). Moreover, Krippl et al. (2020) could demonstrate that by using hybrid modeling, different tangential flow filtration operational modes can be described from the same training data set. More details about recent advances in using hybrid modeling for bioprocess development can be found here (Narayanan et al., 2019, Narayanan et al., 2020; Noll and Henkel, 2020; Bayer et al., 2020b; Narayanan et al., 2021b).

While the hybrid modeling approach is assumed to be able to facilitate improved model performance, the additional utilization of the iDoE concept enables the description of more than one CPP combination, thereby potentially accelerating design space characterization by reducing the number of practical experiments. The combination of iDoE with a hybrid modeling approach for mammalian cell culture has not reported so far. Therefore, we assessed the applicability of hybrid modeling to transfer process knowledge of a Chinese hamster ovary (CHO) cell cultivation derived from a 300 ml bolus feeding shake flask process to a 15 L continuous feeding stirred-tank bioreactor (1:50). Herein, we evaluated the necessary factors to develop a reliable hybrid model structure, which can estimate key process parameters (viable cell concentration (VCC) and product titer) based on the CPPs and amino acid consumption patterns. This hybrid model was presented with new data from 15 L DoE and iDoE cultivations to test its performance. Consecutively, the same hybrid model structure was utilized to build a model based on the iDoE cultivations (re-estimating the model parameters), testing the general applicability of iDoE for mammalian bioprocesses to enable accelerated process characterization in futures studies. The presented applications of hybrid modeling, for transferability and in combination with testing the general applicability of our chosen iDoE setup, have the potential to reduce the experimental effort compared to a static DoE. This facilitates the possibility of saving time, raw materials, and financial resources. Simultaneously, the newly gained understanding of the process is stored in the model in a sustainable and easily retrievable way, so that future work can already be built on this knowledge, independent of expert skills.

## 2 Materials and Methods

### 2.1 Cell Line and Product

A recombinant monoclonal CHO cell line, generated by the Rosa26 bacterial artificial chromosome expression strategy (Zboray et al., 2015), was utilized, producing an antitumor necrosis factor alpha IgG1 (Antibody Lab GmbH, Austria). The cell line was cultured in a chemically defined cell culture medium (Dynamis AGT, A26175-01, Thermo Fisher Scientific, United States) supplemented with 8 mM l-glutamine. The feed medium (CHO CD EfficientFeed A, A1442001, Thermo Fisher Scientific, United States) was supplemented with 0.1% (v/v) antifoam C (A8011, Sigma-Aldrich, Germany) and additional 10, 20, or 30 g L^−1^ d-glucose and 7 g L^−1^ l-asparagine monohydrate.

### 2.2 Design Space

**FIGURE 1:**
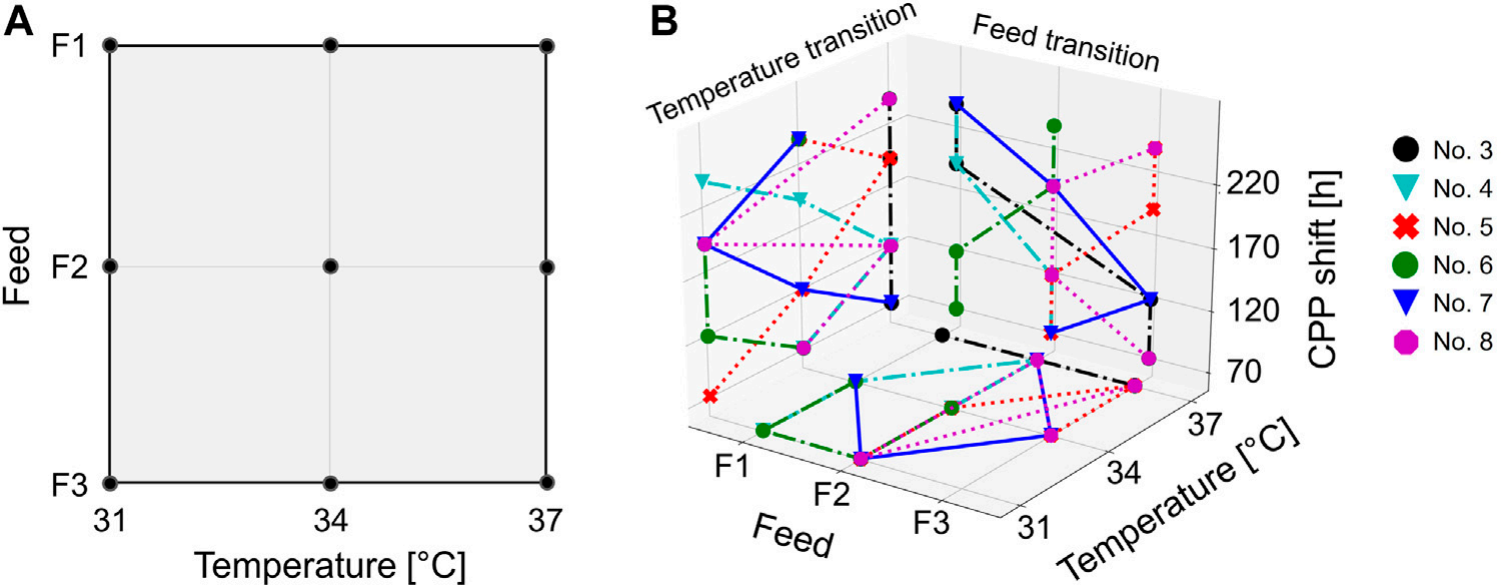
Design space of the DoE performed in a shake flask and 15 L scale. The DoE was conducted as a two-dimensional three-level full-factorial setup comprising the cultivation temperature and the glucose concentration in the feed **(A)**. The CPP shifts of the intensified bioprocesses (15 L) were performed according to the iDoE approach **(B)**. Herein, the CPP transitions are displayed as additional planes (*z*-axis). The individual iDoE bioprocesses are represented by different colors and symbols.

With the cultivation temperature and the glucose concentration in the feed, two CPPs on three levels were chosen in order to investigate these two factors in the design space. Due to the lack of knowledge about the impact of iDoE, only these simple process parameters were chosen. The cultivation temperature was varied between 31°C, 34°C, and 37°C, and additional 10, 20, or 30 g L^−1^ glucose was added to the existing feed medium. These three medium variations are subsequently referred to as F1, F2, and F3, respectively. The harvest criterion for all experiments was set to a viability threshold of <70%. With respect to the different performed bioprocess scales, it is not to be expected that known scale-up issues are influencing the bioprocesses, e.g., mass-transfer limitations and concentration gradients (Marques et al., 2010; Noorman, 2011). The significant difference between shaker-scale and bioreactor-scale experiments was found in the respective executed DoE approaches. For the shaker scale, a simple full-factorial design was performed. On the bioreactor scale, besides static experiments (one CPP combination per cultivation), a more complex iDoE approach was conducted (see also Tables 1 and 2). This intensified approach consisted of several intra-experimental CPP shifts. The herein-captured design space slightly differed, since in the iDoE approach, an extreme CPP value combination was not characterized as indicated in Figure 1. This particular CPP combination was omitted in the 15 L scale because high glucose concentrations in the supernatant of the shake flask experiments were observed (up to 15 g L^−1^). This finding indicated unfavorable conditions for cell metabolism and undesirable settings for upcoming processes.

**TABLE 1:** Used process parameters and run numbers for the 18 shake flask DoE experiments. F1, F2, and F3 represent the amount of added glucose in the feed: 10, 20, and 30 g L^−1^, respectively.

No	Process parameters from the start of fed batch (72 h)	
	Temperature (°C)	Feed
1	31°C	F1
2		
3	31°C	F2
4		
5	31°C	F3
6		
7	34°C	F1
8		
9	34°C	F2
10		
11	34°C	F3
12		
13	37°C	F1
14		
15	37°C	F2
16		
17	37°C	F3
18		

**TABLE 2:** Used parameters and time points of CPP shifts in iDoE experiments in 15 L stirred-tank bioprocesses.

No	DoE mode	Intra-experimental parameter shifts			
		Start of fed batch (72 h)	Shift at 120 h	Shift at 192 h	Shift at 240 h
1	static	36.3°C/F3	—	—	—
2	static	34°C/F1	—	—	—
3	intensified	37°C/F3	—	37°C/F1	—
4	intensified	34°C/F2	37°C/F2	34°C/F1	31°C/F1
5	intensified	31°C/F2	34°C/F2	37°C/F3	34°C/F3
6	intensified	34°C/F1	31°C/F1	31°C/F2	34°C/F2
7	intensified	37°C/F2	34°C/F3	31°C/F2	34°C/F1
8	intensified	34°C/F3	37°C/F2	31°C/F2	37°C/F3
9	static	34°C/F2	—	—	—
10	static	34°C/F2	—	—	—
11	static	34°C/F2	—	—	—

### 2.3 Shaker-Scale Experiments

Eighteen shaker-scale fed-batch experiments with bolus feed (3.3% v/v per day) were conducted as duplicates in shake flasks (working volume of 300 ml), resulting in a full-factorial characterization of the two-dimensional design. For all 18 experiments, the seeding density was set to 2.5 × 10^5^ cells ml^−1^, and the batch phase was conducted at 37°C for 3 days. Available online data comprised the cultivation temperature, CO_2_ content, and the rpm of the shaker platform. The temperature shift was conducted at the start of the fed-batch process. In Table 1, this experimental setup is displayed. A more detailed explanation of the operating procedure and used substances can be found in Sissolak et al. (2019).

### 2.4 Bioreactor-Scale Experiments

The process design was scaled up to a 15 L stainless steel stirred-tank reactor (LabQube Bilfinger Industrietechnik Salzburg GmbH, Austria). Eleven experiments were conducted on the bioreactor scale, of which five were performed with static CPP combinations and six with the intensified operating mode (intra-experimental CPP shifts). Available online data comprised the cultivation temperature, inlet process air, inlet CO_2_ to control the pH, dissolved oxygen, stirrer speed, O_2_, and CO_2_ in the off-gas. Herein, the iDoE approach was performed to investigate the general applicability of CPP shifts to characterize more than one CPP combination per experiment and understand the impact on specific rates for subsequent modeling. In accordance with the shaker-scale experiments, for all 11 experiments, the seeding density was 2.5 × 10^5^ cells ml^−1^, and the batch phase was conducted at 37°C for 3 days. As before, the first parameter shift was conducted at the start of the fed-batch process. The experimental setup and the conducted intra-experimental CPP shifts to capture multiple CPP combinations in the design space within one experiment are displayed in Table 2. The cultivation temperature was shifted by changing the set point temperature. Shifts for the glucose concentration in the feed were enabled by connecting feeds with the same medium constituents but different amounts of glucose (F1, F2, or F3) to the reactor. Depending on the current CPP setting, the respective feed was supplied to the reactor. A more detailed explanation of the operating procedure and the used chemicals can be found in Pappenreiter et al. (2019).

### 2.5 Offline Analytics

The total cell concentration was determined by counting the cell nuclei using the particle counter Z2 (Beckman Coulter, United States). The VCC was determined by assessing the culture viability, using a hemocytometer and trypan blue exclusion.

Carbohydrates were determined via ion exclusion chromatography (HPX 87H, 300 × 7.8 mm, #1250140, Bio-Rad, United States) at 25°C and the amino acids via a reversed-phase HPLC (Eclipse Plus C18 column) at 40°C on an Agilent 1200 series (Agilent, United States).

The product titer was determined by bio-layer interferometry (Octet System, QK, ForteBio, United States).

All offline variables were measured once per day during the whole process (day 0 to harvest) and additionally 6 h after each performed CPP shift in the iDoE experiments. More detailed explanations of the used analytical methodology can be found in Pappenreiter et al. (2019) and Sissolak et al. (2019).

### 2.6 Unsupervised Learning

Principal component analysis (PCA) was applied to analyze the potential input variables for the hybrid model concerning available variables in both the shake flask and the bioreactor system. We therefore used the cultivation temperature and offline analytes (glucose and amino acids) of the shaker and 15 L DoE cultivations. PCA was performed to detect latent structures, being accountable for the data variance, and to investigate possible differences between the shaker and bioreactor scale. PCA was performed with MATLAB (2019b, MathWorks, United States), taking scaling of all inputs into account.

### 2.7 Hybrid Model Development

#### 2.7.1 Model Building

As model inputs, the cultivation temperature (°C), glucose (g L^−1^), glutamine (g L^−1^), asparagine (g L^−1^), alanine (g L^−1^), and aspartate-to-glutamate ratio were chosen to estimate the two response variables of primary interest for this study: the VCC (10^6^ cells ml^−1^) and the product titer (g L^−1^). Even though the glucose concentration is of high interest for bioprocesses, it was not selected as a model output since the chosen feeding strategy guaranteed a glucose concentration above a limiting level. The inputs were standardized using the *z*-score. To estimate the response variables, a serial hybrid model structure was utilized. The data-driven model, an artificial neural network (ANN) embedded in the hybrid model, applying a Levenberg–Marquardt regularization algorithm was chosen to estimate the specific growth rate μ and the product formation rate v_p/x_ as propagated estimations for the mechanistic part, as a function of the model inputs (Eq. 1). Nodes of the hidden layer used hyperbolic tangent transfer functions, while the output layer used a linear transfer function.

$$\text{µ},{\text{ v}}_{\text{p}/\text{x}}=f\left(\text{cultivation temperature},\text{ glucose},\text{ glutamine},\text{ asparagine},\text{ alanine},\frac{\text{aspartate}}{\text{glutamate}}\right)$$ (1)

The values derived from the ANN were subsequently used in the mechanistic model, as shown in Eqs 2 and 3), where X is the VCC (g L^−1^), P is the product titer (g L^−1^), and D is the dilution rate (h^−1^) to describe the ratio between the flow of the feed addition into the reactor (L h^−1^) and the overall reactor volume (L). The hybrid model structure creation and the model evaluation were performed in the Hybrid Modeling Toolbox (Novasign GmbH, Vienna, Austria).

$$\frac{\text{dX}}{\text{dt}}=\text{ µ}\cdot \text{X}-\text{D}\cdot \text{X}$$ (2)

$$\frac{\text{dP}}{\text{dt}}={\text{ v}}_{\text{p}/\text{x}}\cdot \text{X}-\text{D}\cdot \text{P}$$ (3)

#### 2.7.2 Model Validation

For validation of the model performance, cross-validation was performed, i.e., the training data were split into a training and a validation partition (random data partitioning). The initial model was built on this training partition, and the parameters were optimized until a minimal error in the validation partition was found. For the shake flask DoE, this split ratio was set to 0.6 and repeated 40 times to generate a sound number of individual models. In the iDoE modeling for each training/validation distribution, one cultivation was always used as validation partition. Model training stopped once no further improvement was observed. A single hidden layer with four neurons delivered the best performance with respect to the normalized root mean square error (NRMSE) (Eq. 4), where y is the analytical value, ŷ is the estimated counterpart for each sampling point (t), $\bar{y}$ is the mean of the analytical values, and N the total number of observations.

$$\mathrm{NRMSE}\left[\%\right]=\frac{\sqrt{\frac{1}{N}\cdot \sum {\left(y_{\left(t\right)}-{\hat{y}}_{\left(t\right)}\right)}^{2}}}{\bar{y}}\cdot 100$$ (4)

#### 2.7.3 Model Averaging

Averaging of the individual models was performed to avoid overfitting. Selecting a single model from each of the cross-validation partitions and averaging over the selected partitions represent a robust way to deal with model uncertainties. To access this final averaged model performance, the NRMSE was used along with the standard deviation (SD) (Eq. 5) and the confidence interval (CI) (Eq. 6), where ŷ_average_ is the estimation of the averaged model, ŷ_model_ is the estimation of the respective model, i the index of these models, and n is the number of observations for each time point.

$$SD_{\left(t\right)}=\sqrt{\frac{1}{n-1}}\cdot {\sum \left({\hat{y}}_{\mathrm{average}\left(t\right)}-{\hat{y}}_{\mathrm{mod}\mathrm{el}{\left(i\right)}_{\left(t\right)}}\right)}^{2}$$ (5)

$${\text{CI}}_{\left(\text{t}\right)}=\;{\hat{y}}_{\text{average}}\pm {\text{SD}}_{\left(\text{t}\right)}$$ (6)

These final averaged hybrid models were applied to the independent test set (external validation), to assess the performance on new data and to investigate the risk of estimation uncertainty.

## 3 Results

### 3.1 Investigation of Scale-Dependent Bioprocess Behavior

**FIGURE 2:**
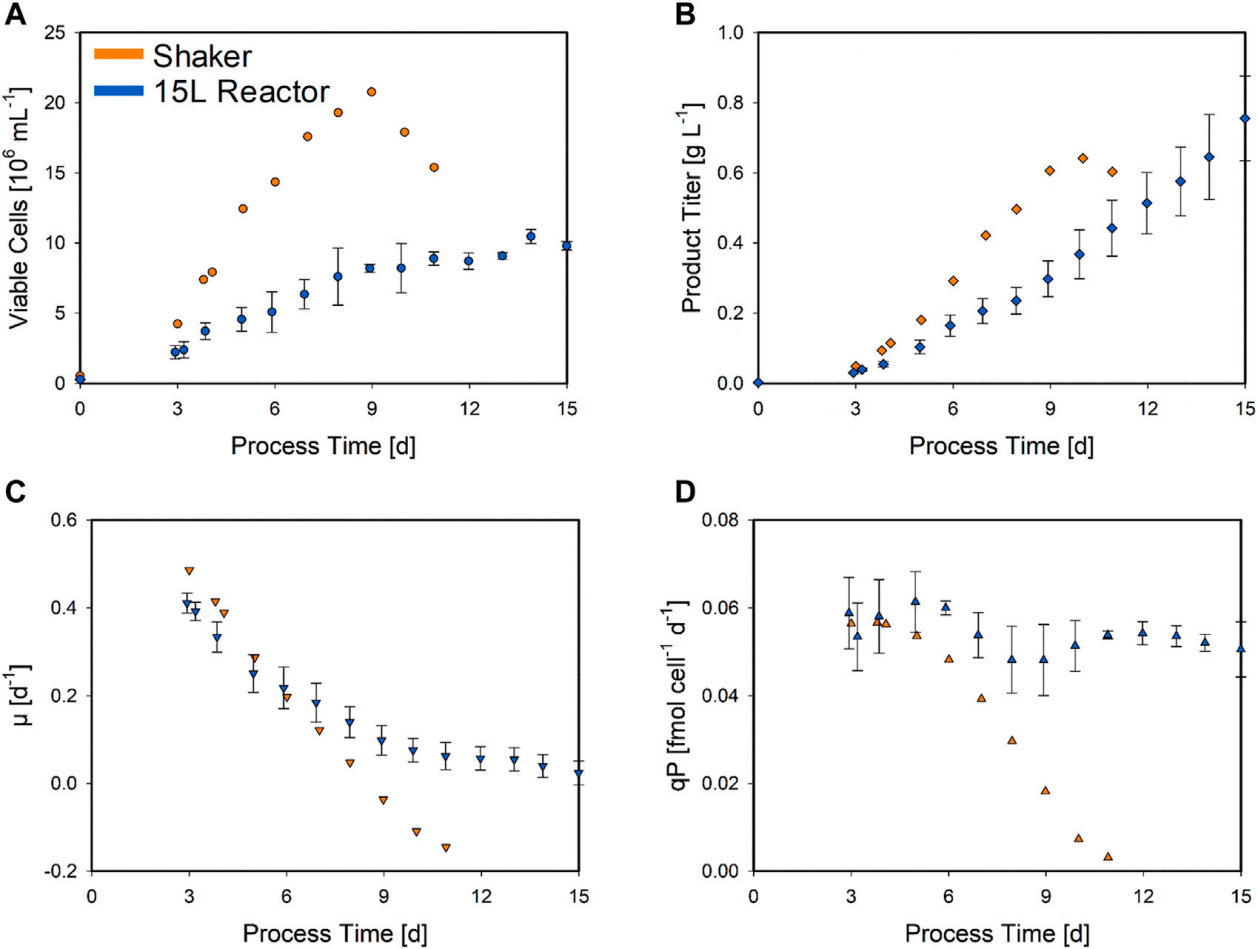
Biomass and product-related trends for the center point CPP combination, performed in the bolus-fed shaker and bioreactor at which a continuous feed was applied. The means of the analytical shaker (orange symbols) and bioreactor (blue symbols) measurements are compared. The average values for the VCC (circles) **(A)**, product titer (squares) **(B)**, specific growth rate (downward triangles) **(C)**, and specific product formation rate (triangles) **(D)** are shown. The standard deviation for the bioreactor experiments (N = 3) is indicated by error bars.

For the development of a transferable hybrid model, it is of high importance that the model is able to understand and describe the metabolic behavior across the different process scales and applied feeding profiles. To shed light on this scale-dependent process behavior, the DoE center point was utilized (CPPs: 34°C and F2). Herein, the VCC and the product titer of the duplicate (shaker) and triplicate (15 L) cultivations of this CPP combination are compared in Figure 2.

Although the two different cultivation process types in Figure 2 were performed with identical CPP settings (DoE center point), significantly different trends were observed. Overall, the bolus-fed shake flask cultivations were terminated after 11 days, as the cell viability fell below the threshold. This was not a problem in bioreactor cultivations applying a continuous feed, where no decrease in viability was observed until the end of the process on day 15. In addition, the VCC trends also differed with respect to the highest values with a maximum of 20.8 × 10^6^ cells ml^−1^ on day 9 in the shake flask culture. This is approximately twice the maximum value in the bioreactor at the end of the process (10.5 × 10^6^ cells ml^−1^) (Figure 2A). However, the maximum product titer of 0.64 g L^−1^ in the shaker reached on day 10 was lower than the maximum value of 0.76 g L^−1^ achieved in the bioreactor at the time of harvest, despite higher cell numbers (Figure 2B). The specific growth rate in the shaker almost linearly decreased from the start until the end (µ = 0.49 to −0.14 day^−1^). The progression of the specific growth rate in the bioreactor started similarly, but this decrease slowed down at day 6 (µ = 0.22 day^−1^), maintaining a positive growth rate until the end (Figure 2C). The same trend was observed for the specific product formation rate (Figure 2D). The shaker again displayed decreasing values, resulting in a negative product formation rate in the end. In contrast, even though the calculation displayed a high standard deviation, the product formation rate in the bioreactor appears to be almost constant, only slightly decreasing. Both these rates were calculated by a cubic smoothing spline function (Bayer et al., 2020a). This comparison demonstrates significant and explicit differences in the process behavior between the shaker scale and the 15 L bioreactor. Additionally, the applied feeding strategies, resulting in different substrate and feeding profiles, may also have a major impact. To also capture this bioreactor-scale-specific behavior, the three 15 L bioreactor center point cultivations were also included in the hybrid model development along with the shaker DoE, enabling the hybrid model to learn the scale-dependent process dynamics.

### 3.2 Input Selection Procedure for Hybrid Model Development

**FIGURE 3:**
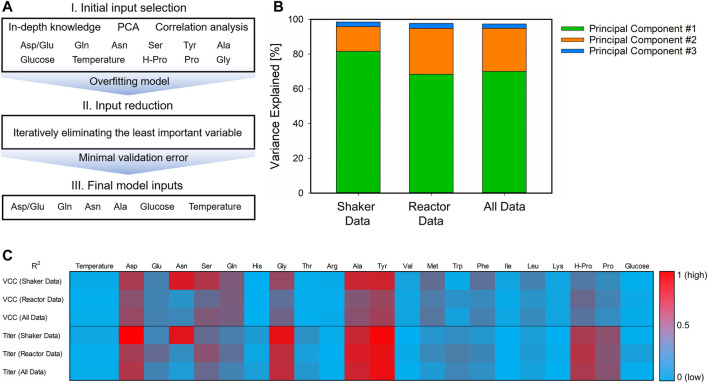
Workflow for the identification of the final hybrid model inputs. Starting with an initial input selection derived from availability and in-depth knowledge, unsupervised learning, and correlation to model targets **(A, I)**, the complex overfitted model was simplified by eliminating the least important variable **(A, II)** until the minimal validation error is found and the final model inputs were identified **(A, III)**. The detailed results for the PCA **(B)** and the correlation heatmap of the target variables **(C)** are presented for the different data sets. For PCA, the explained variance for each principal component is given as stacked bars, and the R^2^ is indicated from low (0, blue) to high (1, red).

The utilization of appropriate inputs for the hybrid model development is essential for robust and transferable performance. For this initial selection of potential variables and to subsequently identify the final model inputs, a workflow consisting of three serial steps was utilized. The individual steps of this workflow are presented in Figure 3. To select inputs, which are of importance for all investigated scales, this input selection framework was performed with three data sets: the shake flask experiments, bioreactor experiments, and all experiments. Here, the bioreactor data comprise the DoE and iDoE experiments since their results were highly similar when separately investigated.

Three different approaches were considered for the initial input selection and the consecutive selection steps (Figure 3A, step I). According to Pappenreiter et al. (2019), the aspartate-to-glutamate ratio indicates metabolic changes in the course of the process and is thus an important process variable. The PCA of the three different data sets displayed comparable results with respect to the principal components. In all three scenarios, two components were already sufficient to explain >94% of the variance in the data (Figure 3B). Herein, glutamine, asparagine, aspartate, serine, and the two CPPs (cultivation temperature and glucose concentration in the feed) were important variables explaining this variance. These were therefore also taken into account for the initial selection. Additionally, more potential model inputs were identified by correlation analysis (Figure 3C). Here, aspartate, glycine, alanine, tyrosine, and hydroxyproline were found to be correlated to the target variables in all data sets. Proline was not considered due to its collinearity to hydroxyproline. With all these inputs, potential overfitting of the hybrid model is reasonable. To avoid this behavior, the inputs to the model were reduced one at a time, and the error was evaluated (Figure 3A, step II). This procedure was repeated until the minimum validation error was found, and therefore, the final hybrid model inputs were identified (Figure 3A, step III). These final inputs comprise the two CPPs, the aspartate-to-glutamate ratio, glutamine, asparagine, and alanine.

### 3.3 Hybrid Model Performance on 15 L DoE and iDoE Bioprocesses

**FIGURE 4:**
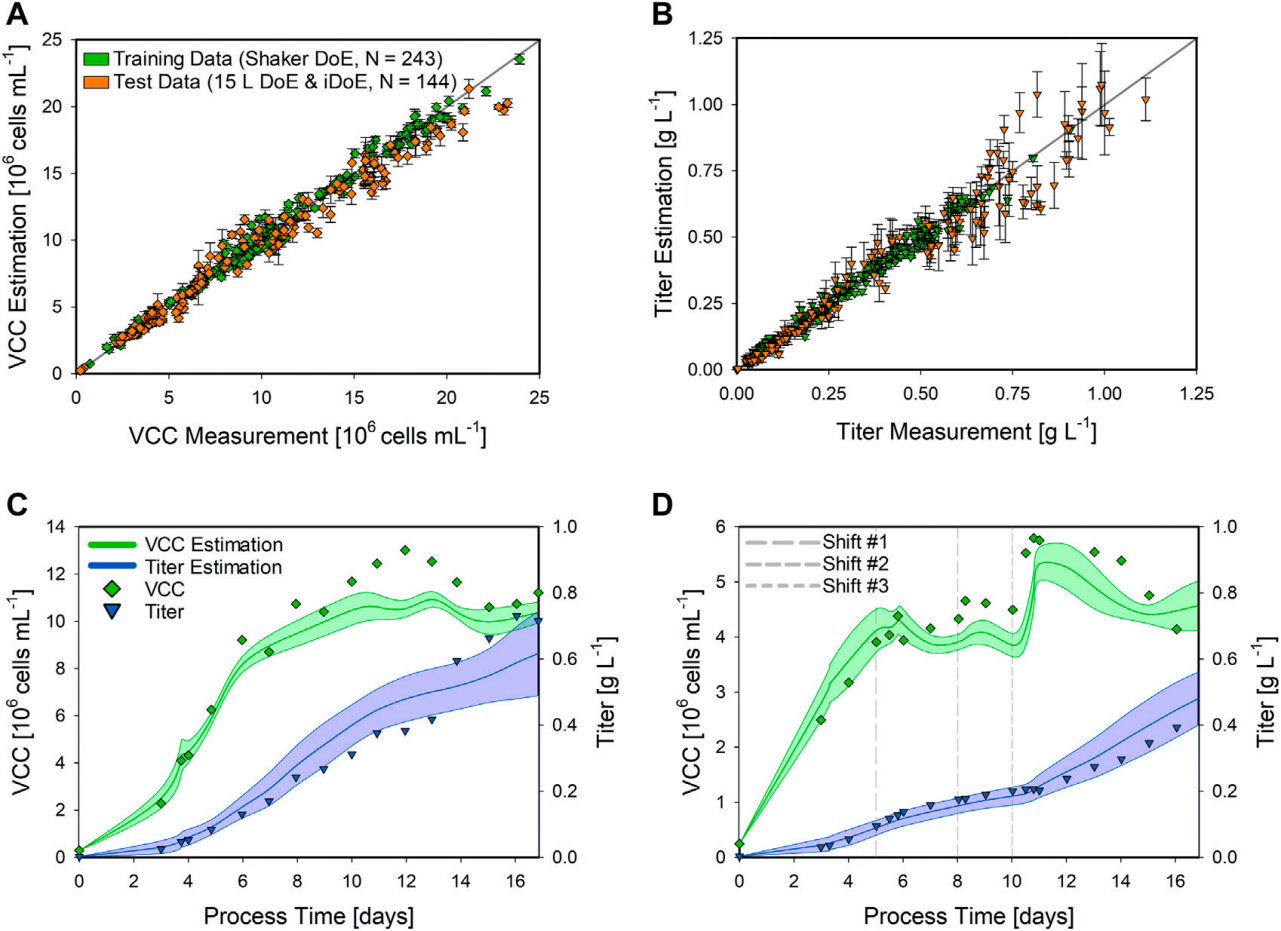
Performance of the shaker hybrid model estimating the VCC and product titer of 15 L bioprocesses. The scatter plots of the hybrid model on the training data (green) and the test data (orange) for the VCC **(A)** and the product titer **(B)** are presented along with the standard deviation as error bars. Detailed time-resolved model estimations are presented for a static DoE **(C)** and an iDoE bioprocess **(D)**. The model estimations for the VCC (green lines) and product titer (blue lines) are indicated along with the respective CI (shaded area). The analytical measurements are given for the VCC (green squares) and the product titer (blue triangles). For the iDoE bioprocess, the time point of the CPP shifts is indicated (dashed grey lines).

Based on the shaker DoE (Table 1) and the three additional center point bioprocesses (Figure 2), a hybrid model was developed utilizing the identified inputs (Figure 3), hereafter referred to as training data. This hybrid model was applied to the 15 L bioreactor runs (Table 2), hereafter referred to as test data, to test the transferability performance. This test set contained two static runs, one at the DoE corner (maximum CPP settings) and the other at intermediate settings (34°C and F10), as well as six iDoE bioprocesses to investigate the robustness and applicability of the hybrid model. Since the hybrid model is trained solely on bioprocesses with static CPP conditions, it was assumed that the iDoE bioprocesses with the intra-experimental CPP shifts would be challenging due to the unseen process behavior. This performance on the test set is presented in Figure 4, displaying the scatter plots for the VCC and the product titer along with time-resolved modeling results for an exemplary DoE (Table 2, No. 2) and an iDoE bioprocess (Table 2, No. 6). The performed CPP shifts for the presented iDoE process are shown in Table 2.

The expanded shaker hybrid model displayed good performance for estimating the VCC in the 15 L bioprocesses (Figure 4A). Overall, the training data were estimated with an NRMSE of 5.53%, while the test data resulted in an NRMSE of 10.92%. For the product titer (Figure 4B), NRMSE values of 8.04% in the training and 17.79% in the test data were obtained. This is also indicated by the more widely spread data in the scatter plot and increased CIs, especially at higher values, which were not present in the training data. This higher standard deviation is also visible in the more detailed time-resolved estimation of the product titer (Figure 4C). Moreover, the hybrid model correctly estimated the trend for the VCC and only displayed inaccurate estimations from day 10 to day 14, not completely matching this peak. The estimation of the product titer of the iDoE bioprocess (Figure 4D) was highly accurate and also displayed the impacts of the CPP shifts, especially visible after the last shift on day 10. While the overall hybrid model performance displayed good transferability, the impact of these conducted CPP shifts was visible in the estimation of the VCC, making the bioprocess interpretation more difficult due to the never-seen bioprocess behavior in the training data.

### 3.4 15 L iDoE Hybrid Model Performance on Static 15 L Bioprocesses

**FIGURE 5:**
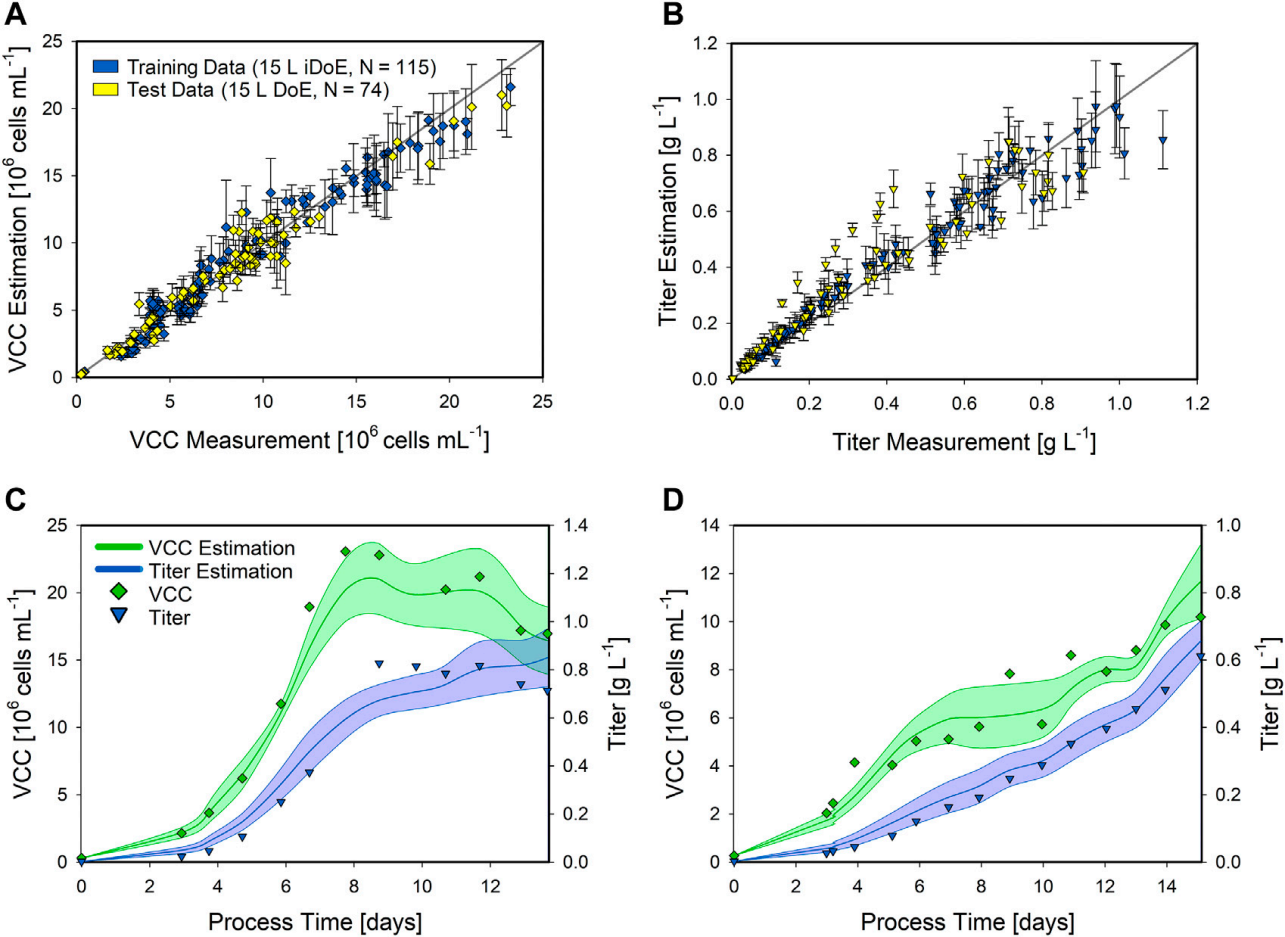
Performance of the 15 L iDoE hybrid model estimating the VCC and product titer of static 15 L bioprocesses. The scatter plots of the hybrid model on the training data (blue) and the test data (yellow) for the VCC **(A)** and the product titer **(B)** are presented along with the standard deviation as error bars. Detailed time-resolved model estimations are presented for two static DoE bioprocesses **(C,D)**. The model estimations for the VCC (green lines) and product titer (blue lines) are indicated along with the respective CI (shaded area). The analytical measurements are given for the VCC (green squares) and the product titer (blue triangles).

Additionally, besides testing the transferability of the identified hybrid model structure, the general applicability of iDoE was of high interest due to its potential of accelerating process characterization by reducing the overall experimental effort. Therefore, a hybrid model based on the 15 L iDoE bioprocesses was developed, hereafter referred to as training data. Since the input selection framework (Figure 3) displayed comparable results for all data sets, the same structure as that for the shaker data was utilized. The hybrid model performance was tested on the static DoE runs, hereafter referred to as test data, presented as scatter plots, and for two time-resolved exemplary bioprocesses (Table 2, No. 1 and No. 10) in Figure 5.

The developed 15 L iDoE hybrid model overall performed well in estimating the target variables, as presented in the scatter plots for the VCC (Figure 5A) and the product titer (Figure 5B). Compared to the more extensive shaker hybrid model, the NRMSE overall increased along with an increased standard deviation, i.e., the VCC was estimated with an NRMSE of 12.16% in the training data and 13.75% in the test data, while the NRMSE for the product titer resulted in 15% (training) and 21.13% (test). The model estimation of the presented static bioprocess, performed at the corner of the DoE (highest temperature and highest glucose concentration in the feed), was close to the analytical values. However, the CI of the VCC was increased compared to that of the shaker hybrid model (Figure 5C). A similar model performance was observed for the second presented bioprocess, performed at the DoE center point (Figure 5D). While the product titer was estimated highly accurately, the estimation of the VCC overall matched the experimental values but displayed a high CI. These results and the overall performance of the hybrid model based on iDoE data indicate the general applicability of iDoE for the modeling of mammalian bioprocesses. Nevertheless, more aspects of iDoE and its proper planning should be investigated in future studies. Thereby, a comparison to DoE will be allowed as well as an increased information yield, i.e., to benefit from the time saved by reducing the number of experiments while maintaining high explanatory power of the model.

## 4 Discussion

In this case study, we demonstrated that for the transferability of a bioprocess, the required characterization of the process behavior is challenging due to highly different scale characteristics as well as differences in the feeding strategies. Even though the same CPP settings were applied, significant differences between shake flasks (300 ml) and the laboratory scale (15 L) were observed (Figure 2). The herein-presented different trends for key process variables and their respective rates again demonstrate that process transferability is not straightforward, and challenges are found early on. These varying process trends may derive from the reactor design or the different feeding strategies, i.e., bolus feed in the shaker and the continuous linear feed in the bioreactor. An approach to still describe and understand the behavior during upscaling can be found in hybrid modeling. The advantage of applying hybrid models is that the process behavior can be understood more rapidly, saving experiments. Hybrid modeling has been proven to be beneficial for such complex bioprocessing problems since multiple knowledge sources are combined. Within this work, we wanted to get insights into the cellular behavior in CHO cells and challenge the applicability of hybrid modeling with respect to process transferability and a combined utilization with iDoE. Herein, we only tested the general applicability of such intra-experimental CPP shifts to potentially reduce the required number of practical experiments. This iDoE approach was investigated in the 15 L scale because the execution of such intra-experimental CPP shifts is not executable in shake flask experiments (considering only one available incubation system).

To develop robust process models, meaningful inputs and the correct data structure are crucial (Figure 3). Unsupervised learning (Figure 3B), correlation to the target variables (Figure 3C), and whether available previous in-depth knowledge proved to be highly valuable for the initial selection of potential inputs, which displayed importance and were available for all investigated scales. This workflow enables the reduction from the initial 11 inputs down to 6, which were identified to be relevant in all performed DoEs: the two CPPs, the aspartate-to-glutamate ratio, glutamine, asparagine, and alanine, which are also confirmed by literature to be of high impact for CHO cultivations (Carrillo-Cocom et al., 2015; Fan et al., 2015; Zhang et al., 2016; Ghaffari et al., 2020). Due to this procedure, the final hybrid model could be applied for both scales and is more generic and its performance more robust when presented with new data.

We first tested model transferability performance of the developed shaker hybrid model extended with the center point triplicates. We then applied the model to the static DoE and iDoE bioprocess data in the 15 L scale (Figure 4). Herein, the estimation of the VCC was close to the analytical values (Figure 4A), while the product titer estimation displayed decreased accuracy and increased CIs along with rising values (Figure 4B). These model uncertainties were probably due to product formation kinetics in the bioreactor, which was never observed in the shaker scale. The NRMSE was within the range of the analytical error, further indicating an adequate model performance. The time-resolved examples for a static (Figure 4C) and an intensified bioprocess (Figure 4D) confirmed the applicability of the shaker-scale model for the 15 L scale. This behavior indicates that the basic metabolism, CPP impacts, and amino acid consumption patterns can be understood and properly interpreted by the shaker-scale model, even when dealing with data from bioprocesses with intra-experimental CPP variations.

Moreover, the model captured and correctly interpreted the impact of the intra-experimental CPP shifts in the iDoE experiments. Herein, it is also visible that the cells do not rapidly adapt to the new settings, but an adaptation time of roughly 2 days seems essential. Compared to the adaptation time of 1 h reported for microbial systems, the expected shifting frequency must be considered carefully (Bayer et al., 2020b). This indicates that longer intervals between the shifts may be beneficial in increasing the knowledge gain from iDoE experiments. Nevertheless, the hybrid model based on shake flask experiments only extended with the center point triplicate was able to understand the bioreactor-scale process behavior in the challenging test set from iDoE experiments. This demonstrates that by choosing appropriate training data in combination with a suitable hybrid model structure, experimental effort on a larger scale can be reduced without losing information and process knowledge.

In regard to the model structure, utilizing additional relevant design variables would potentially increase the transferability since more impacting factors can be considered for modeling, e.g., the center point cultivations to recalibrate the shaker hybrid model possibly could have been avoided. The challenge herein becomes the number of required experiments to capture complex high-dimensional design spaces. Therefore, iDoE might be a suitable choice to facilitate this operational implementation, if properly planned. Furthermore, by expanding the model, using multiple design variables, accurate description of more outputs would be enabled. However, it must be taken into account that the overall modeling error increases if additional outputs are taken into account since each individual error will impact the overall performance.

Second, the direct comparison between a hybrid model trained on iDoE to describe DoE data was investigated. Therefore, the previously identified inputs and the hybrid model structure were used to train a hybrid model based on the 15 L iDoE bioprocesses, and the model was applied on the static DoE data (Figure 5). Herein, the number of performed experiments to potentially screen a design space could be reduced, while the knowledge about the CPP impact on the specific rates is increased, providing an accelerated learning rate for the hybrid model to rapidly understand the process behavior. In comparison to the shaker hybrid model, the scatter plots show increased CIs for both target variables (Figures 5A,B). This could be due to the reduced amount of data for model training and also due to the performed iDoE itself. However, since no prior information about an appropriate iDoE concept for mammalian cells is available by now, these bioprocesses were performed for the first time ever in the frame of the work reported here. The higher CIs are also seen in the time-resolved examples (Figures 5C,D), especially for the VCC, which indicates that the lowered amount of data results in increased model uncertainties. This herein-reduced information further highlights the importance of planning such an iDoE properly for process modeling and process characterization. Additionally, to exclude an irreversible impact of previous CPP settings on the cells, the so-called memory effect, is a crucial consideration and of high importance for the iDoE approach. To understand and investigate the potential occurrence of this memory effect, when setting up an iDoE in future studies, experiments should be performed with the same experimental conditions but in opposite directions. The impacts of the process change magnitude and the ideal time point of the changes, i.e., earlier or later in the process, were not considered yet. Finally, the already-stated shifting frequency must be considered. Definitely, this promising concept of performing intra-experimental shifts of CPPs must be further investigated to maximize its potential. Nevertheless, the iDoE hybrid model performed well, also considering the limited input factors available from the shaker cultivation, demonstrating the applicability of the concept for CHO cells.

## 5 Conclusion

We could demonstrate that a hybrid model trained on 300 ml bolus feeding shake flask DoE could be used to correctly estimate the cell behavior and product formation in a 15 L stirred-tank bioreactor with a continuous feed, within the same design space, requiring only minimal recalibration. Furthermore, we could show that the information content from iDoE experiments in the 15 L scale is comparable to that from DoE experiments. Therefore, iDoE can potentially be used to cover a specific design space with fewer experiments compared to classic DoE approaches. However, a lot of attention has to be drawn to design the iDoE experiment in a clever way to extract the maximum information per process time. Finally, due to the limited available variables in the shake flask experiments (merely offline analytes), the established descriptive hybrid models are applicable to gain process understanding and capture the process behavior but cannot be used for process control. Granted that more monitoring and control strategies are already available in the small scale, the created models could potentially be used for multiple control purposes (pH, amino acids, and glucose). These different setups would enable an even better understanding and transferability and finally enable real-time monitoring and control opportunities.

We could demonstrate that a hybrid model trained on 300 ml bolus feeding shake flask DoE could be used to correctly estimate the cell behavior and product formation in a 15 L stirred-tank bioreactor with a continuous feed, within the same design space, requiring only minimal recalibration. Furthermore, we could show that the information content from iDoE experiments in the 15 L scale is comparable to that from DoE experiments. Therefore, iDoE can potentially be used to cover a specific design space with fewer experiments compared to classic DoE approaches. However, a lot of attention has to be drawn to design the iDoE experiment in a clever way to extract the maximum information per process time. Finally, due to the limited available variables in the shake flask experiments (merely offline analytes), the established descriptive hybrid models are applicable to gain process understanding and capture the process behavior but cannot be used for process control. Granted that more monitoring and control strategies are already available in the small scale, the created models could potentially be used for multiple control purposes (pH, amino acids, and glucose). These different setups would enable an even better understanding and transferability and finally enable real-time monitoring and control opportunities.

## Data Availability

The raw data supporting the conclusion of this article will be made available by the authors, without undue reservation.

## Abbreviations

ANN, artificial neural network; CHO, Chinese hamster ovary; CI, confidence interval; CPP, critical process parameters; CQA, critical quality attributes; DoE, design of experiments; iDoE, intensified design of experiments; NRMSE, normalized root mean square error; PCA, principal component analysis; QbD, quality by design; SD, standard deviation; VCC, viable cell concentration.
